# Sport, and use of anabolic androgenic steroids among Icelandic high school students: a critical test of three perspectives

**DOI:** 10.1186/1747-597X-5-32

**Published:** 2010-12-20

**Authors:** Thorolfur Thorlindsson, Vidar Halldorsson

**Affiliations:** 1 University of Iceland, v/Sudurgata, 107 Reykjavik, Iceland

## Abstract

**Background:**

This study investigates the use of anabolic androgenic steroids (AAS) among a national representative sample of high school students in Iceland. We test several hypotheses drawn from three perspectives. The first perspective focuses on the use of AAS as an individual phenomenon motivated by the desire to succeed in sport. The second perspective views the use of AAS as shaped by norms and values embedded in social relationships of formally organized sport. The third perspective suggests that factors outside sport, which have been shown to correlate with the use of other substances, predict the use of AAS.

**Method:**

We use logistic regression and predicted probabilities to analyze data from a national representative survey of 11031 Icelandic high school students.

**Results:**

Our results indicated that the use of AAS is not significantly related to participation in formally organized sports. However, it positively relates to fitness and physical training in informal contexts. We found a relatively strong relationship between the use of AAS and the use of illicit substances and a moderate relationship between AAS use and alcohol and tobacco consumption. We also found a significant negative relationship between AAS use and school integration and school achievement, and a significant positive relationship between AAS use and school anomie. The relation between AAS use and family-related variables was weaker. Finally, we found that the relationship between sport participation, physical exercise, and AAS use varies across levels of anomie and integration.

**Conclusion:**

Our findings suggest that the use of AAS and especially illegal substances should be considered more as a social and a health problem rather than a sport specific issue. We found that high school students participating in fitness and informal training outside of formally organized sport clubs are the main risk group and should be the target of prevention efforts. However, this should not be done at the expense of general risk factors that affect AAS and other substances used by the general population. Finally, we suggest that prevention efforts should target both groups and individuals.

## Background

A growing body of evidence indicates that the use of anabolic androgenic steroids (AAS) can be dangerous to overall health. The harmful effects of AAS on the cardiovascular system, mental health, the liver, and the reproductive system are well documented [1-7]. Young people who use AAS face greater health risks compared to other age groups. Some of the consequences of AAS use, such as increased mood swings, aggression, and physical closure, may cause particularly serious physiological, psychological, and social problems for people in this age group [8,9]. The evidence suggests that we need to turn our attention to adolescents and young people to gain a better understanding of AAS use among this particularly vulnerable age group.

The public discussion about AAS use has centered on sport, especially on elite athletes who are considered at an increased risk when it comes to using these agents, presumably because of the strong incentives that entice winners to cheat [7,10-12]. This focus on sport, however, has diverted attention away from the wider social context outside sport, as well as the health and social issues involved. While sport may play an important role in the use of AAS, this problem needs to be considered in a broader context, which includes a host of social and individual factors in addition to individual motivation, fair play, and sport ethics. We suggest three perspectives that help address the broader social context of AAS use among young people.

The first perspective accepts the popular explanations for young people's AAS use, focusing on sport participation as a key factor. It sees AAS use among youths as an individual level problem [13,14] and portrays it as a rational and intentional act where the athlete systematically calculates the benefits and the risks before making a decision. The individual who takes the risk faces the consequences on his own. Using AAS to enhance performance in sport is tempting because athletic success paves the way to fame and fortune. This perspective assumes that the explanation is to be found in the utilitarian motives of the self-interested, rationally calculating individuals shape human behavior in an attempt to maximize their gain. Athletes are considered to be at an increased risk when it comes to using these agents because of the strong incentives to cheat. The world around young people emphasizes success. Success in sport brings popularity, admiration, and approval from peers [15]. Furthermore, it can be argued that those who aspire to become professional athletes are tempted to take AAS to enhance their performance in order to increase their prospects of establishing a career in professional sport.

Prior research suggests that it may be critical to distinguish between different categories of physical exercise [16]. Comparing young people who engage in physical activity, irrespective of the social organization in which they train, with those that do not do any physical exercise does not adequately test the sport and AAS hypothesis. The theories that propose that sport is the key to AAS use should focus on organized competitive sport embedded in formal institutions. In Europe, this means sport practiced in sport clubs. In some other parts of the world, such as the USA, the emphasis is more on school-related sports. In both cases, sport is formally organized with systematic training and competition in mind. Those who engage in physical activity outside of formal institutions are not bound by rules of fair play and other norms and values that define the competitive sport clubs. They do not have the same incentives to win as those who compete regularly in formally organized sport events. It is also critical to distinguish between committed athletes who train regularly - i.e. four times a week or more - and those who train with less intensity, for example, once or twice a month. In the same vein, we argue that young people who train intensively in formally organized sport clubs are more likely to use AAS than athletes who train intensively outside the organized sport environment.

This utilitarian concept of the social actor can be contrasted with the "social relations" approach from classical sociology. From this perspective, norms and values anchored in the structure of social relationships are the factors that shape human behavior [17]. Purposive action is seen as embedded in a concrete ongoing system of social relations [17]. Actors do not behave like isolated individuals outside the social context, nor do they follow a prewritten social script determined by their membership in a particular group or social category. They are substantially influenced by their involvement in ongoing social relationships. Therefore, athletes act as social actors embedded in social relationships determined by the social organization of sport. Participation in formally organized sport means that one is bound by rules of fair play and other norms and values that define competitive sport. There are, however, certain sport subcultures, such as in professional cycling and baseball, where the use of performance enhancing drugs seems to be widespread [18,19]. Using AAS violates the code of fair play and equality that are a central characteristic of competitive sport. The penalties for steroid use in sport are harsh and include disqualification, stripping away medals, erasing records, and banning from competition. Prior research that has shown a negative relationship between sport and the use of tobacco, illicit drugs, and in even alcohol supports this view only indirectly [20-22]. In short, these two sport-specific perspectives make different predictions about the relationship between sport participation and the use of AAS among young people.

The third perspective that captures AAS use among young people does not focus on sport. It postulates that factors other than sport participation influence the use of AAS among youth and adolescents. It implies that the social institution of sport overlaps with other social activities outside sport. Thus, social factors outside sport may influence the use of AAS among youth inside and outside sport in a similar way to how they influence the use of other substances. Recent research indicates that the use of AAS among adolescents correlates with the use of other substances and thus lends weight to this view [3,23,24]. Illicit substances, alcohol, tobacco, and AAS may be clustered together because the same social factors influence their use [22]. Durkheim's work, especially his work on suicide [25] where he develops his theory of anomie and integration, inspired the development of many of these social factors to begin with. The core idea of anomie theory is that ambiguity in rules and goals increases a sense of meaninglessness and reduces commitment to conventional institutions. Anomie may be particularly pertinent to adolescence, which is a period in life where individuals are struggling with life's purpose and meaning while seeking acceptance and a sense of self-worth [26]. Durkheim's theory of integration underlines the involvement with and attachment to family, religion, and central social institutions. Some scholars have emphasized that attachment to parents and key social institutions, such as family, religion, and school, commitment to conventional norms and values, academic achievement, the desire for autonomy, religiousness, meaninglessness, family structure, parent education, social support, and social control all play a role in young people's delinquent behavior and substance use [22,27-30]. This "socially inclusive" perspective implies that the same social variables that predict delinquency and substance use also predict the use of AAS among young people.

The use of AAS among athletes provides an interesting test of the utilitarian and relational perspectives of the social actor. These two popular perspectives make different predictions about the sport-AAS relationship. The utilitarian perspective predicts that AAS use should be more common among athletic youths because participation in organized sport provides the motives for using AAS. The sport-specific social relations perspective implies that participation in organized sport should not increase AAS use. The third perspective, the "social inclusive perspective," predicts that social factors determine AAS use among youths and adolescents who participate in sport as well as those who do not. We do not argue that the two perspectives are mutually exclusive. They may in fact overlap in several ways. One particularly theoretically relevant example of such a relationship is Durkheim´s theory of social integration and anomie, which suggests that the balance between the utilitarian and the social relations perspectives varies empirically according to the state of anomie and integration [25,26]. The utilitarian aspects of society are more salient when the state of anomie is high rather than low. Likewise, the utilitarian aspects of society are stronger in a society characterized by high rather than low integration. This means that the relationship between sport and the use of AAS should vary across levels of anomie and integration. It should be stronger when anomie is high and weaker when anomie is low. The reverse should hold for integration.

In this paper, we use a nationally representative sample of 11031 high school students to test several hypotheses about the use of AAS drawn from the three different perspectives discussed above. We also go beyond prior research by clarifying definitions of sport participation and by distinguishing between various types of physical activity and sport participation. In short:

• We estimate the use of AAS for the high school population at the national level.

• We compare AAS use across social categories of sport and physical exercise with AAS use among those who are physically inactive.

• We investigate the relationship between several social risk factors and AAS use.

• Finally, we look at the variation in the relationship of sport participation and informal physical exercise with AAS use across levels of social integration and anomie.

## Methods

### Participants and procedures

The data used for analysis came from a 2004 national survey of Icelandic high school students. The sample is nationally representative, consisting of all students attending high school in Iceland. All data collection was conducted in strict accordance with the Privacy and Data Protection Authority in Iceland. The Icelandic Centre for Social Research and Analysis conducted the survey. Teachers administrated anonymous questionnaires to all students who were present in class on October 20, 2004. The students received instructions that included the purpose of the study, and were told that some of the questions they would be answering dealt with sensitive issues. The students had 80 minutes (two high school lessons) to complete the questionnaires. Upon completion, each student placed and sealed their questionnaire in a blank envelope. A total of 11,031 students participated in the survey. There were 5279 (48.4%) males and 5617 (51.6%) females in the study. The numbers for gender do not match the number of participants due to missing values. A question on how often one had used *Relevin *(a fake drug) was used to discard ambiguous data, removing 113 questionnaires from the study. Finally, 10918 questionnaires were used with 5195 (48.2%) males and 5585 (51.8%) females. The average age was 17.7 years (median = 17; range 15-24; SD = 1.84).

### Measures

AAS use was measured on a scale from never to 40 times or more. An attempt was made to make the question simple, direct and unambiguous, to avoid overrepresentation [31]. The question was: How often in your lifetime have you used AAS (Icelandic: "anabólíska stera"). The variable was coded as never used AAS = 0 and used AAS = 1.

Sex was codes as females = 0; males = 1.

Age was measured by year of birth, with the youngest participants born in 1990 and the oldest born before 1980. Year of birth was then replaced by actual age at the time of the study.

#### Sport participation variables

Informal sport or physical activity (such as for instance fitness) was measured on a scale from never to almost every day. Participation in formally organized sport clubs was measured on a scale from never to 6 times per week or more. The measures of formal and informal sport participation were mutually exclusive. No subjects who participated in informal sport participated in formally organized sport in the sport clubs. As we have pointed out in the background discussion, the way sport participation is categorized is of critical importance. To make sure that our analysis covered that problem adequately, both participation in formally organized sport clubs and informal sport participation were recoded in three different ways. Participation in formal sport therefore included three variables. Sport 1 variable included those who participate 1x a week or less = 0; those who participate 2x a week or more = 1. Sport 2 included those who participate less than 1x a week = 0; those who participate 1-3x a week = 1; those who participate 4x a week or more = 2. Sport 3 included those who participate 1x a week or less = 0; those who participate 4x a week or more = 1. The same was done for participation in informal sports.

#### Social factors

Alcohol use was measured by how often one had been drinking in the last 30 days on a scale from never to 40x or more. The variable was recoded into a dummy variable (never drunk in the last 30 days = 0, sometime drunk in the last 30 days = 1).

Tobacco use consists of smoking cigarettes, using spit tobacco, and using snuff tobacco. Cigarette use was measured on a scale from none in the last 30 days to more than 20 cigarettes a day. The variable was recoded into a dummy variable (less than daily = 0, daily smoking = 1). Spit tobacco and snuff tobacco were both measured on a scale from never to 40x or more. They were recoded into dummy variables (not used in the last 30 days = 0, used in the last 30 days = 1).

Four questions assessed how often participants used illegal substances such as hashish, amphetamines, LSD or Ecstasy, on a scale from never to 40x or more. All variables were recoded into dummy variables (never used = 0, used = 1).

#### Family variables

Parents' education was measured for each parent on a 5 point ordinal scale from less than high school to completing a university degree coded as little education = 1, medium education = 2, higher education = 3.

Family structure was coded as 0 for those living with both parents and 1 for other arrangement.

Parents' support consisted of 4 response categories for the 5 variables measured: warmth and caring, talking about personal subjects, school advice, other advice, and general assistance, assessing how difficult or easy it was to receive support from parents measured on a 5 point ordinal scale ranging from very difficult to very easy. The variable was then recoded into a 3-point scale ranging from very difficult to get support = 1, to moderately easy/difficult to get support = 2, to very easy to get support = 3 (Cronbach's Alpha = .87).

Parents' rule setting consisted of 5 response categories assessing rules of domestic behavior, rules about coming home at night, parents knowing with whom their children were at night, parents' knowledge of where their children were at night, and parents knowing their children's friends, which were measured on a 5-point ordinal scale ranging from does not apply to do apply very well. The variable was then recoded into the following categories: slack rule setting = 1, moderately slack/strict rule setting = 2, strict rule setting = 3 (Cronbach's Alpha = .81).

#### School variables

Grades were measured for the following subjects: Icelandic, Math, English, and Danish. The scale for each subject consisted of 8 response categories ranging from under 4 to 10. The grades for the four subjects were then combined into a GPA score for each respondent. The GPA variable was then recoded into low grades = 1, medium grades = 2, high grades = 3.

School integration was measured using 5 items measured on a 5-point scale from almost always applies to almost never applies. The questions were: I feel that I am not prepared for class; I feel that I do not put enough work in my studies; I want to change schools; I feel that I do not belong in the school; and I feel that I am being mobbed at school. The variable was then recoded into weak school integration = 1, medium school integration = 2, strong school integration = 3 (Cronbach's Alpha = .61). This reliability is below the desirable .70 mark. The findings that involve the school integration scale may therefore result in underestimation of the relationships involved.

#### Anomie

Two scales were used to measure anomie. Each one of these scales captures different aspects of the concept. The general state of anomie was measured using a 6-item ordinal scale developed by Dean [32] and adopted by Thorlindsson and Bernburg [26]. The response categories ranged from strongly agree to strongly disagree. The items included: most rules can be broken if they do not apply; I follow my own rules; there are very few absolute rules in life; it is difficult to trust anything; no one knows what is expected of him in life; and you can never be certain of anything in life. The variable was then recoded into low anomie = 1, medium anomie = 2, high anomie = 3 (Cronbach's Alpha = .78).

School anomie was measured on an ordinal scale developed by Thorlindsson and Bernburg [26]. The scale emphasizes the meaninglessness of anomie and consists of three items: I feel that my studies have no meaning; I want to quit school; and I don't get along with my teachers. The variable was then recoded into low school anomie = 1, medium school anomie = 2, high school anomie = 3 (Cronbach's Alpha = .61). This reliability is lower than the reliability reported in earlier studies (see Thorlindsson and Bernburg, [26]). It is also below the .70 mark. The findings that involve the school anomie scale may therefore result in underestimation of the relationships involved.

Institutional trust was measured on a 9-item ordinal scale that consists of 4 response categories ranging from very much to very little trust. The scale measures trust in the following institutions: the church, primary school, high school, the court system, the police, the government, the media, and the trade union. The variable was recoded into low institutional trust = 1, medium institutional trust = 2, high institutional trust = 3 (Cronbach's Alpha = .85).

Religion was measured on a 4-item ordinal scale, each item assessed using 5 response categories ranging from almost never to more than once a week. The 4 items included; goes to church, participates in religious work, prays to God, and reads the Bible or other books. The variable was recoded into low religion = 1, medium religion = 2, high religion = 3 (Cronbach's Alpha = .68).

#### Analysis

We used both logistic regression and predicted probabilities to analyze the data. The coefficients reported for the logistic regression are odds ratios, where each odds ratio is the multiplicative effect of a 1-unit change in the independent variable on the odds of the dependant variable. The value of the odds ratios may be highly sensitive to the way the variables are divided into categories. This may cause problems, especially when variables do not have clear naturally or theoretically defined categories. To overcome this limitation, we also present our results in terms of predicted probabilities, treating the independent variables as if they were continuous variables. Presenting the relationship between AAS and selected variables as predicted probabilities illustrates the nature of the relationships (such as linearity) and complements the odds ratios analysis.

## Results

Table 1 shows the use of AAS by gender, age, participation in organized sport, and leisure/fitness, indicating that the use of AAS is low since only 0.9% of participants have ever used AAS.

**Table 1:** Demographies and selected sport participation variables by AAS use (percentages and numbers)

	Have used AAS	
	%	Number
All*	0.9	94
Male	1.6	81
Female	0.2	12
Youngest group (15-16 yrs.)	0.3	10
Middle group (17-18 yrs.)	0.6	23
Oldest group (19-24 yrs.)	1.7	51
Oldest males	3.4	46
Oldest females	0.3	4
**Sport participation in sport club**		
Less than 1x week	0.9	61
1-3x a week	0.6	7
4x a week or more often	1.1	21
**Fitness sport participation**		
Less than 1x week	0.8	47
1-3x a week	0.4	15
4x a week or more often	2.3	27

*N = 10918

Gender differences are striking as 1.6% of boys have used AAS compared to 0.2% of girls. The proportion of students who have used AAS increases significantly with age (t = 6.180, df = 10213 p = .001).

Table 1 also shows that the proportion of AAS use is slightly higher among athletes who participate in organized sports 4x a week or more compared to participants who do not practice formal sports. The difference, however, is not significant (t = .733, df = 8984, p = .463). The group that reports the proportionally highest AAS use consists of individuals who participate in leisure/fitness sports outside sport clubs 4x a week or more.

Table 2 shows the results of the logistic regression with steroid use as a dependent variable adjusted for gender and age. The table shows the odd ratios and the 95% confidence interval for different categories of formally organized sport and recreational physical exercise, use of other substances, and some social risk factors.

**Table 2:** Logistic regression model for AAS use by selected social variables adjusted for gender and age (odds ratios and 95% confidence intervals)

	Have used AAS	
	OR	(95% CI)
**Sport participation in sport club**		
Variable 1 (Less than 1x; 2x or more)	1.125	.685-2.516
Variable 2 (Almost never; 1-3x; 4x or more)	1.103	.840-1.449
Variable 3 (Less than 1x; 4x or more)	1.331	.783-2.262
**Fitness sport participation**		
Variable 1 (Less than 1x; 2x or more)	1.444	.828-2.516
Variable 2 (Almost never; 1-3x; 4x or more)	1.468**	1.096-1.967
Variable 3 (Less than 1x; 4x or more)	2.665***	1.583-.4.486
Odds ratio for steroid use among those who practice sports 4x a week or more in sport clubs and outside sport clubs.	2.27***	
**Tobacco and Alcohol Use**	3.154**	1.490-6.677
Daily smoke	1.754*	1.055-2.916
Other tobacco use in the past 30 days	1.612**	1.207-2.154
Drunk in the past 30 days	1.765*	1.024-3.043
**Illegal substance use**	4.891***	3.007-7.953
Used hashish	3.977***	2.463-6.422
Used amphetamines	7.487***	4.661-12.027
Other substances	7.815***	4.868-12.546
**Family**		
Parents education	.900	.660-1.228
Living with parents	.964	.503-1.846
Parents support	.781	.602-.1.014
Parents rule setting	.863	.484-1.540
**School**		
Grades	.559**	.369-.846
School Anomie	1.448**	1.124-1.867
School Integration	.732*	.559-.691
**Other**		
Anomie	1.167	.635-2.145
Institutional trust	.960	.540-1.705
Religion	.939	.825-1.068

*Significant at p < 0.05 level

** Significant at p < 0.01 level

*** Significant at p < 0.001 level

p-values were from Wald t-tests, df = 1

The results reveal that the differences between those who participate in organized sport and those who do not participate in organized sports are not significant. Moreover, the results also show that there are no significant differences between athletes who participate 4x a week in organized sport and non-athletes. In other words, the findings do not support the utilitarian perspective, which holds that formally organized sport provides the key to steroid use among young people. It is noteworthy that those who participate in recreational exercise and fitness outside formally organized sport 4x a week or more are almost 2.7 times more likely to use AAS compared to those who do not participate in formal sports. Furthermore, the findings in Table 2 indicate that athletes who participate in formally organized sports are significantly less likely to use AAS than individuals who participate in recreational physical exercise or fitness outside the sport clubs. The findings consistently indicate that it is not participation in formally organized sport, but rather participation in informal leisure and fitness that predicts steroid use.

**Figure 1:**
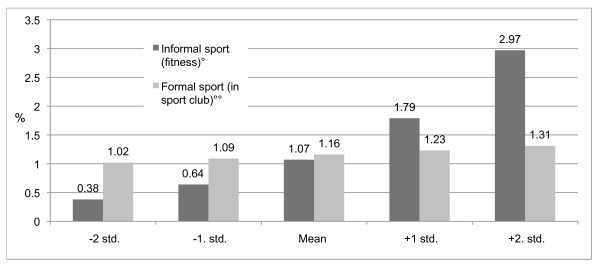
**Predicted probabilities of AAS use by informal and formal sport participation, in percentages, adjusted for gender and age**. ° (Wald = 12.74, df = 1, p = .001). °° (Wald = .303, df = 1, p = .582)

Figure 1 shows predicted probabilities of AAS use for both formal and informal sports from -2 standard deviations up to +2 standard deviations. The figure shows that the relationship between informal sport/fitness participation and steroid use is fairly strong and increases in a linear fashion. The predicted probabilities of sport participation in sport clubs, on the other hand, remain relatively more constant as sport participation increases. In fact, the predicted probabilities of steroid use increase only slightly with increased participation in formal sport.

Table 2 also shows a relatively strong relationship between steroid use and the use of other substances, as predicted. The findings summarized in this table indicate that those who smoke daily are about 1.8 times more likely to use AAS compared to those who do not smoke. A similar relationship is obtained for spit tobacco and alcohol. Regular use of both tobacco and alcohol increases the risk of steroid use significantly. The strong relationship between steroid use and the use of illicit substances is noteworthy.

**Figure 2:**
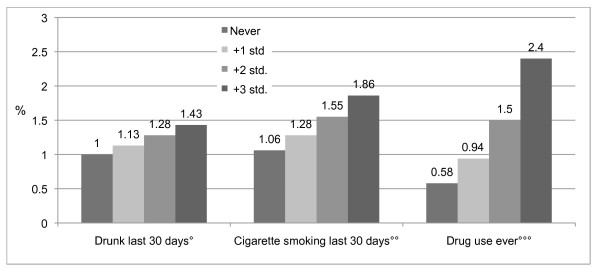
**Predicted probabilities of AAS use by alcohol, tobacco and illicit drug use, in percentages, adjusted for gender and age**. °(Wald = 1.393 df = 1, p = .238). °°(Wald = 3.844, df = 1, p = .005). °°°(Wald = 69.940, df = 1, p = .001)

We also used various substances as continuous variables predicting substance use at different points in the distribution. Again, the findings confirm our previous analyses. The results shown in Figure 2 depict how the use of AAS increases in a linear fashion in relation to the use of illicit substances and smoking. The figure thus reveals a moderate (alcohol) to a fairly strong (illicit substances) relationship between use of various substances and steroids. However, we must bear in mind the low numbers of subjects in some groups. The relationship between AAS use and other drug use and cigarette smoking are significant at the .05 level, while alcohol use is not, as shown in Figure 2.

These findings lend considerable support to the social context perspective because they show a strong relationship between the use of AAS and the use of other substances.

Table 2 also presents the logistic regression analyses for AAS use across different social background factors that relate to substance use according to previous research. While all of the relationships are in the predicted direction, only the three variables pertaining to school are significant. Thus, getting good grades reduces the odds of using AAS almost in half, whereas school anomie increases the odds of using AAS by almost 50%.

**Figure 3:**
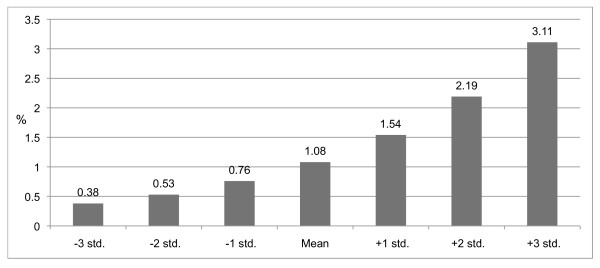
**Predicted probabilities of AAS use by school anomie°, in percentages, adjusted for gender and age**. °(Wald = 13.674 df = 1, p = .001)

**Figure 4:**
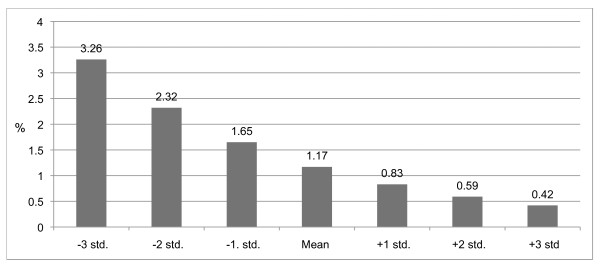
**Predicted probabilities of AAS use by grades in school°, in percentages, adjusted for gender and age**. °(Wald = 8.747, df = 1, p = .003)

Using the social variables as continuous variables indicated that the risk of using AAS increases in a linear fashion. Figures 3 and 4 clearly reveal significant relationships of school anomie and grades, underlining the importance of the school environment.

**Figure 5:**
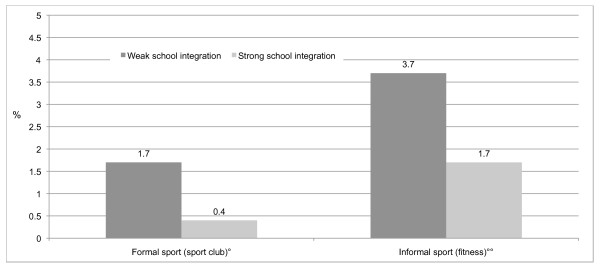
**AAS use in percentages by school integration within formal sport and informal sport**. °Confidence intervals at the .05 level for formal sport: 1.46-1.94 (weak school integration), 0.28-0.52 (strong school integration). °°Confidence intervals at the .05 level for informal sport: 3.35-4.05 (weak school integration), 1.46-1.94 (strong school integration).

**Figure 6:**
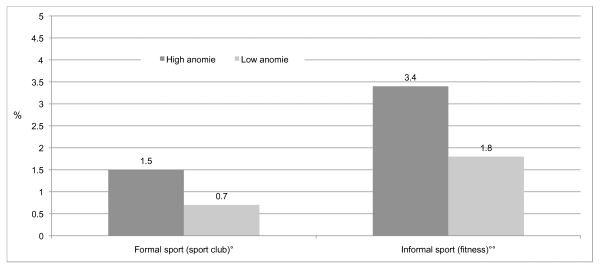
**AAS use in percentages by school anomie within formal sport and informal sport**. °Confidence intervals at the .05 level for formal sport: 1.27-1.73 (high school anomie), 0.54-0.86 (low school anomie). °Confidence intervals at the .05 level for informal sport: 3.06-3.74 (high school anomie), 1.55-2.05 (low school anomie).

For those who train 4 times or more a week, figures 5, and 6 further reveal that there is a relationship between AAS use within and outside formal sport clubs according to the level of school integration and school anomie.

These findings are in line with the theoretical perspective, which suggests that the relationship between sport and the use of AAS is stronger when anomie is high and weaker when anomie is low, and stronger when integration is low and weaker when integration is high.

## Conclusion

The findings above indicate that the use of AAS is not a widespread problem among Icelandic high school students in general. In fact, the use of AAS among Icelandic high school students is considerably lower than the use of illegal substances such as cannabis or amphetamines [33]. While these findings are in line with other research among young people in Scandinavia [23], the figures for the USA are considerably higher [3,34].

The findings above indicate that the use of AAS is unevenly distributed across gender and age. They thus support earlier findings suggesting that steroid use is a "male thing" [3,14,34]. Interestingly, our findings regarding girls' use of AAS are quite close to those of Kanayama et al. [31]. The corresponding figures for female and male athletes show a similar trend. Gender predicts steroid use much better than participation in formally organized sport. These findings support the suggestions made by Stewart and Smith [14] that social factors related to masculinity may influence the use of steroids among athletes. Unfortunately, we did not have any direct measures of masculinity, macho values, or other such factors; therefore, we cannot pursue the issue further. This issue, however, should be addressed in future research.

The two sport-specific perspectives receive little empirical support. Participation in organized sport, characterized by formally organized competition, does not predict the use of AAS among young people in Iceland. While it may be noted that the relationship between AAS use is stronger for those who are more committed and train harder, the relationship is not significant. In this context, it is important to note that high school students who participate in physical exercise and fitness outside formally organized sport constitute the group most likely to use AAS. This group of students is much more likely to use AAS than those who engage in formally organized sport. The findings hold for high school students who participate in these two different social contexts four times a week or more. The comparison between formally organized sport and informal training provides a critical test. Steroids are used to increase muscle strength and thus enhance performance. Strength can be a desirable outcome of physical training in more than one context. By comparing two groups committed to physical training in two different social contexts, the formal and the informal, we can isolate the influence of the formal social organization of sport on the group level. Our findings underscore the importance of clearly defining sports. Participating in formally organized sport means that one is bound by rules of fair play and other norms and values that define competitive sport. We need to make a clear distinction between formally organized sport, which takes place under the supervision of a coach in a very structured environment, and physical exercise or a leisure activity, which takes place in a more unstructured environment. The latter is not bound by the same set of norms and values that define the institution of formally organized sport. We must also bear in mind that formally organized sport makes efforts to control and prevent the use of AAS among its members, whereas informally organized physical activity, such as fitness training, does not. The ideal sport ethics of equity and fair play combined with drug testing may counteract the temptation to use AAS.

This does not mean that we have identified individual level motives. We recognize that the relationship between levels of analysis can be quite complex (for a discussion of motives on different levels of analysis see Chen and Kanfer [35]). We can only say that the desire to win in formal competition is not widespread enough among high school students participating formally organized sport to set it apart from other groups in society. In contrast, the use of AAS is much higher among the group that participates informal physical exercise, where physical strength and muscle building is a desirable quality. Thus, our findings suggest that AAS are used more to enhance appearance than to enhance sport performance. Prior findings indicate that the motives for using AAS and the paths that lead to steroid use may be diverse among individuals who engage in fitness training and bodybuilding. These individuals may decide to use AAS to "enhance their masculinity" in order to elevate social status, or simply to enhance their mood [15,24]. It is an important limitation of this study that we lack both direct measures of fair play and other important norms that characterize formally organized sport and measures of individual motivation. Including such measures in future studies could increase our understanding of the interplay of group level and individual level processes in the development of substance use among young people.

The third perspective, which emphasizes the wider social context, receives considerable empirical support in our findings. We found a significant relationship between the use of AAS and the use of other substances, such as alcohol, tobacco, and illicit drugs. It is worth noting that we obtained the highest odd ratios for the relationship between steroid use and illicit drug use, which is in line with earlier findings [16,23,34]. Thus, the use of AAS among young people is to some extent a part of multi-drug behavior. The question is to what degree this multi-drug behavior is rooted in the wider social environment.

Our findings regarding the relationship between social factors and AAS use cast some light on this. All of the relationships tested are in the direction predicted. However, the family variables, such as social support, monitoring, and parents' education, which have been shown to be systematically related to substance use among Icelandic adolescents [22], are not significantly related at the .05 level. One reason for the weak relationship between the family variables and steroid use in our study may be that the high school students in our sample are older than the adolescents in prior studies. It makes sense to argue that parental influence diminishes, as the children grow older. Our findings indicate that compared to the use of other substances, the use of AAS emerges later in life or at an age when parents are starting to have less influence. Three of the school-related variables tested - school grades, school anomie, and school commitment - are significantly related to AAS use at the .05 level. These findings underscore both the importance of the school environment and the process of integration and anomie in the life of young people.

The findings show that the relationship between sport participation and AAS use varies significantly across levels of school anomie and school integration. As predicted, the relationship between sport and AAS use is stronger for those adolescents who experience anomie and weaker for those who are better integrated into the school environment. These findings are of significant theoretical and practical interest because they demonstrate how the social institution of sport overlaps with other social activities and institutions. The importance of the school environment for this particular age group is noteworthy.

The findings above indicate that it is not sufficient to focus on organized sport as the key to understanding AAS use among high school students. Our tendency to view the problem as specific to sport may distort the picture considerably. In order to understand the use of AAS among young people, we have to put it in a wider context and treat it more as a social or public health issue. AAS use is primarily a male problem; it is highly related to the use of other drugs and predominantly affects high school students who train outside of formally organized sport environments. It is influenced by risk factors outside of the sport environment, especially the school environment.

Youth sport has provided a specific platform for discussing AAS use among young people. Our findings suggest that this emphasis is too narrow. In fact, they suggest that the school provides a more important social context for preventive efforts against AAS use than organized sport. But organized sport may still have the potential to become a major breeding ground for AAS use in the future. The institution of sport overlaps with society in many ways, and the boundaries are often unclear. Sport may therefore have to defend its key missions, become a more salient part of the prevention efforts against the use of AAS, and strengthen its key defining characteristics against outside influences. Youth sport is considered to promote core social values and help integrate youths into mainstream society [22]. Using AAS violates the core idea of fair play and the notion that sport should promote the health and wellbeing of children and youths. Goldberg et al. [36] have shown that the use of AAS, as well as the use of alcohol and illicit drugs, can be prevented with a team-centered education. In fact, they concluded that athletic teams in schools provide an optimal context for drug prevention and health education [36,37]. Including AAS in sport-oriented prevention could prevent AAS use from becoming an accepted part of sport achievement.

Focusing on the sport and school environment does not attend to AAS use among young males who engage in fitness and recreational physical activities outside formally organized sport. Our findings suggest that this group is a major risk group for the use of AAS. Reaching this group may be hard because it operates in an informal social context, which is not part of the school or the sport environment. A second implication is that even though it is important to isolate special risk groups, it is of equal importance to work with general risk factors that influence the use of several substances simultaneously. The use of AAS among high school students coincides with the use of other substances, and is influenced by social factors that have been shown to predict the use of other drugs. These facts underscore the importance of adopting a broader, more inclusive view of prevention and substance use. While isolating and targeting special risk groups is important, it should not be at the expense of working with general risk factors that affect the entire population. Finally, our findings suggest that it is important to pay attention to the social context and to direct prevention efforts at groups as well as individuals.

## Competing interests

The authors declare that they have no competing interests.

## Authors' contributions

The first author, TT, designed the study and made the first draft. The second author, VH, conducted the statistical analysis. Both authors worked on all aspects of the final version.
